# Enhancing Hydrophobicity and Oxygen Barrier of Xylan/PVOH Composite Film by 1,2,3,4-Butane Tetracarboxylic Acid Crosslinking

**DOI:** 10.3390/polym15132811

**Published:** 2023-06-25

**Authors:** Guoshuai Liu, Kang Shi, Hui Sun, Biao Yang, Yunxuan Weng

**Affiliations:** 1 College of Chemistry and Materials Engineering, Beijing Technology and Business University, Beijing 100048, China2030402057@st.btbu.edu.cn (K.S.); ybiao@th.btbu.edu.cn (B.Y.); 2 Beijing Key Laboratory of Quality Evaluation Technology for Hygiene and Safety of Plastics, Beijing Technology and Business University, Beijing 100048, China

**Keywords:** xylan, 1,2,3,4-butane tetracarboxylic acid, hydrophobic properties, barrier properties

## Abstract

Hemicellulose has potential advantages in food packaging because of its abundant reserves, degradability and regeneration. However, compared with fossil-derived plastic films, hemicellulose-based films show inferior hydrophobicity and barrier properties because of their low degree of polymerization and strong hydrophilicity. Focusing on such issues, this work covers the modification of a xylan/polyvinyl alcohol (PVOH) film using 1,2,3,4-butane tetracarboxylic acid (BTCA) as esterifying agent. The thus prepared composite film was more compact owing to the esterification reaction with xylan and PVOH forming a crosslinked network structure and reducing the distance between molecular chains. The results showed that BTCA had a positive effect on the oxygen barrier, hydrophobicity and mechanical properties of the composite film. The tensile strength of the xylan/PVOH composite film with 10% BTCA content increased from 11.19 MPa to 13.99 MPa. A 20% BTCA loading resulted in an increase in the contact angle of the composite film from 87.1° to 108.2°, and a decrease in the oxygen permeability from 2.11 to 0.43 (cm^3^·µm)/(m^2^·d·kPa), corresponding to increase in the contact angle by 24% and a decrease in oxygen permeability by 80%. The overall performance enhancement indicates the potential application of such composites as food packaging.

## 1. Introduction

The depletion of fossil resources and increasing environmental awareness have let to research focusing on sustainability and renewability [1]. The development of biodegradable or bio-based materials from a wide range of renewable sources is currently at the forefront of research. Bio-based materials are made from biomass resources extracted directly from plants or marine organisms, such as polysaccharides and proteins [2]. Polysaccharides have inherent properties such as biodegradability, biocompatibility and non-toxicity [3], facilitating their use in the form of thin films and edible coatings in food packaging. Many naturally occurring polysaccharides such as cellulose [4], hemicellulose, starch and chitosan [5] have been used in the formulation of such films [6].

As the second most abundant polysaccharide in plants, hemicellulose is the main component of the plant cell wall, accounting for 20–35% of the total weight in the cell wall [7]. Hemicellulose is a low-molecular-weight polysaccharide composed of C-6 and C-5 sugar units. It has a varying and complex structure, mostly consisting of branched chains [8]. According to the structural differences, hemicellulose is mainly divided into xylan, mannan, β-glucan and xyloglucan [9]. Xylan is the most common hemicellulose in nature [2], and its main chain [10] is linked to xylose with a 1,4-β glycosidic bond. Most xylans contain acetyl groups, most of which are located on C-3 of xylose, while a few are located on C-4 of xylose. At the same time, 4-O-methyl-α-D-glucuronic acid is linked to xylose residues. As a polar polymer, xylan has good barrier performance to nonpolar molecules (oxygen), and a large number of hydroxyl groups contained in xylan are also conducive to the chemical modification of xylan [11], meaning that it can be developed as a biofilm for food packaging [12]. However, the application of xylan in food packaging is limited because of its short molecular chain, low molecular weight, poor film-forming ability and strong hydrophilicity. Recently, many attempts have been made to solve these defects, including blending xylan with other polymers [13,14], adding plasticizers [15,16] and the chemical modification of xylan [17,18], so as to improve the properties of xylan film. Creating composites of xylan and polymers is an effective method to improve the film-forming properties.

Polyvinyl alcohol (PVOH) is a kind of polymer with a linear backbone. With a large number of hydroxyl groups present in its molecular chains, PVOH has high polarity and high hydrophilicity, and is very suitable for blending with natural polymers [19]. PVOH has attracted widespread attention in food packaging because of its good mechanical properties, resistance to organic solvents, biocompatibility and good film-forming properties [20]. There have been reports on the incorporation of PVOH as a co-substrate with natural polysaccharides (cellulose, hemicellulose, chitosan, etc.). Pan et al. [21] used loblolly pine as a raw material, first obtained hemicellulose by means of the alkali extraction-ethanol precipitation method, then used the solution casting method to make the material uniform [22], and prepared a PVOH/hemicellulose composite film with good mechanical properties. Gao et al. [23] prepared a composite film by blending xylan with PVOH. Sorbitol was added as a plasticizer to improve the processability of hemicellulose and the flexibility of the hemicellulose-based composite film.

Protecting food from oxygen and moisture during transportation, handling and storage is usually one property required for food packaging. In this regard, low oxygen permeability and hydrophobicity are important properties of packaging materials [24]. Xylan films are naturally hydrophilic, and their strength decreases after being exposed to moisture [25]. To this end, various methods have been reported to address this issue, which include doping nanoparticles [26] or chemical modification [27] to bring the hemicellulose chains closer and reduce the number of hydrophilic hydroxyl groups. The modified films showed improved barrier effects and hydrophobicities which are comparable to those of traditional petroleum-based packaging materials. Among these efforts, Zhao et al. prepared a hemicellulose-based composite film [28] using sodium trimetaphosphate as a crosslinking agent. Their composite film showed a good barrier effect and hydrophobic properties in addition to excellent mechanical properties.

Because of health concern, formaldehyde-based crosslinking agents are considered unsuitable. Polycarboxylic acids, such as maleic acid [29], citric acid [30], and 1,2,3,4-butanetetracarboxylic acid (BTCA) [31], have been used as crosslinking agents for natural polymers. BTCA has the advantages of low price, safety and non-toxicity. Compared with maleic acid and citric acid, BTCA has four carboxyl groups, which can provide more reaction sites for esterification. In the presence of a catalyst, BTCA can reduce the required reaction temperature and form cyclic anhydride intermediates to complete the esterification reaction [32]. As a non-phosphorus catalyst, sodium propionate has been reported to be used in the esterification between BTCA and starch [33]. Compared with the common catalyst sodium hypophosphite [34], sodium propionate is more affordable and non-toxic to the human body, and thus can be used as a food preservative. BTCA has been used as a crosslinking agent to improve the properties of starch and cellulose [35,36,37], but its effect on the hydrophobicity and oxygen permeability of xylan/PVOH composite films has not been studied.

This paper covers the preparation and properties investigation of a xylan-based film using PVOH as a co-matrix. A safe, water-soluble crosslinking agent, BTCA, was selected to esterify xylan and PVOH to form a crosslinked structure. An environmentally friendly catalyst, sodium propionate, was used to accelerate the reaction, and sorbitol was used as a plasticizer. The xylan/PVOH composite film was prepared by means of the solution casting method. The effects of BTCA on the hydrophilicity, barrier properties, mechanical properties and thermal stability of the composite film were studied.

## 2. Materials and Methods

### 2.1. Materials

Beech xylan (M_w_ is 20,000 to 30,000 g/mole, Shanghai Yuanye Bio-Technology Co., Ltd., Shanghai, China), PVOH (polyvinyl alcohol with a degree of polymerization of 1700 and alcoholysis degree 99%, Shanghai Titan Scientific Co., Ltd., Shanghai, China), D-sorbitol (M_w_ is 182.173 g/mole, Shanghai Yuanye Bio-Technology Co., Ltd., Shanghai, China), BTCA (M_w_ is 234.16 g/mole, Bide Pharmatech Ltd., Shanghai, China), and sodium propionate (M_w_ is 96.06 g/mole, Shanghai Macklin Biochemical Technology Co., Ltd., Shanghai, China) were used as received without further purification.

### 2.2. Preparation of BTCA-Crosslinked Xylan/PVOH Composite Films

PVOH was completely dissolved in deionized water at 95 °C to form a uniform and transparent solution. Then, xylan and sorbitol were added, and the mixture was stirred for 30 min. Afterwards, BTCA of various amounts and 80 mg of sodium propionate were added. The mixture was stirred for 10 h. After the reaction, the solution was cooled to room temperature and sonicated for 20 min. Finally, the solution was poured into a polystyrene Petri dish (13 cm × 13 cm); the bubbles in the solution were removed, and the film was dried at room temperature. In this experiment, the mass of BTCA accounted for 0% to 50% of the total mass of PVOH, xylan and sorbitol. The composition of each film was shown in Table 1. Before all measurements, the dried films were placed in an oven at 25 °C for 48 h.

### 2.3. Analytical Methods

Fourier transform infrared spectral (FT-IR) analysis was performed with a infrared spectrometer (Nicolet LSJ20, Waltham, MA, USA) with scanning range of 440 to 4000 cm^−1^, 32 scans, and a resolution of 4 cm^−1^.

For scanning electron microscope (SEM) analysis, the film sample was cut into a small square piece, and a thin layer of gold was sputtered on the surface. The surface morphology of the film was observed with a scanning electron microscope (Quanta 250 FEG, FEI, Hillsboro, OR, USA) using an acceleration voltage of 10 kV.

The X-ray diffraction (XRD) analysis of xylan, PVOH and xylan/PVOH composite films was performed by using an X-ray diffractometer (D8 Advance, AXS, Bruker, Karlsruhe, Germany). The diffraction angle (2*θ*) varied in the range of 10° to 80° at a scanning speed of 5°/min.

The contact angle of the films was measured using a contact angle measuring instrument (OCA35, Data Physics Instruments, Charlotte, NC, USA). Water droplets with volumes of 2 μL were dropped on the surface of the film. The instrument was used to capture the image of the droplet, and the contact angle was calculated from the image. Five different positions of each sample were measured, and the average value was reported as the contact angle of the sample [38].

According to China national standard GB/T 1038-2000 (2000), the oxygen permeability of the film was measured with a differential pressure gas permeameter (VAC-V2, Labthink, Jinan, China) after the film sample was cut into slices with a diameter of 10 cm. Measurements were performed at 23 °C under a relative humidity of 50%. The average value of 3 samples was taken.

According to standard GB/T1040.2-2006 (2006), the film was cut into a rectangular sample of 10 mm × 80 mm. The thickness of the sample was measured using a thickness gauge. A microcomputer-controlled electronic universal testing machine (CMT6104, MTS Systems Co., Ltd., Shanghai, China) was used to test the tensile properties of the samples at a tensile speed of 5 mm/min with the distance between the clamps set at 60 mm. Five rectangular specimens were cut from each film. The tensile strength and elongation at break of the film were the average of the test results of the 5 rectangular specimens.

Thermogravimetric analysis (TGA) was conducted on a thermogravimetric analyzer (STA7200, Hitachi, Tokyo, Japan) in a temperature range of 40 °C to 600 °C with a heating rate of 20 °C/min and a nitrogen flow rate of 20 mL/min.

## 3. Results and Discussion

**Figure 1:**
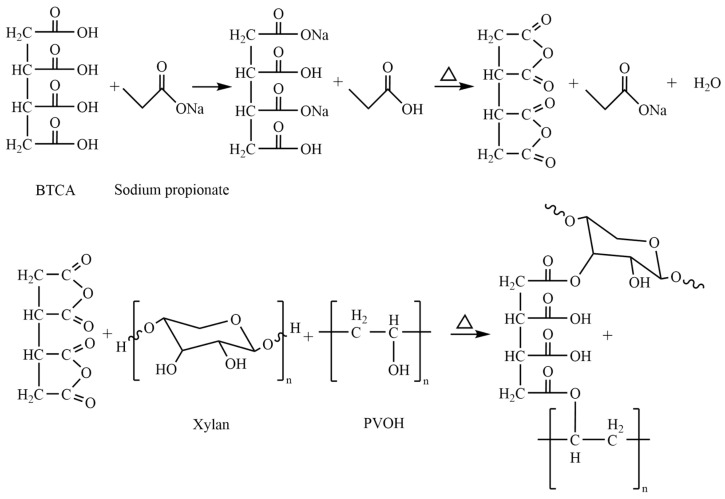
Crosslinking reaction of the matrix (PVOH-Xylan) and BTCA.

BTCA can dehydrate two adjacent carboxyl groups to form cyclic anhydride intermediates under the catalysis of sodium propionate. The intermediate of anhydride was esterified with hydroxyl groups in xylan and PVOH. Therefore, the network crosslinking structure of xylan/PVOH composite film can be formed by using BTCA as a crosslinking agent, as shown in Figure 1.

### 3.1. Structural Analysis

**Figure 2:**
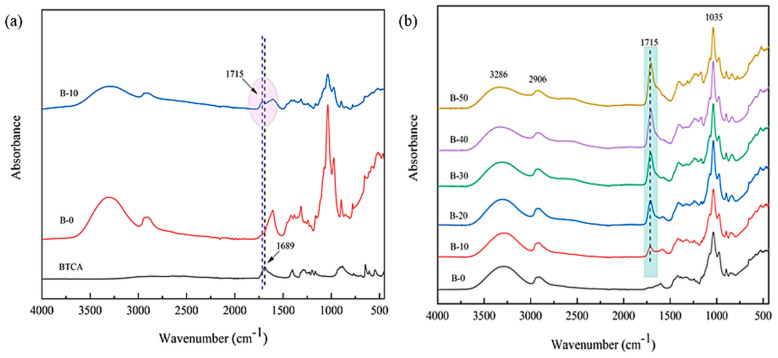
FT−IR spectra of BTCA (**a**) and BTCA-crosslinked xylan/PVOH composite film (**b**).

The FT-IR spectra of crosslinked xylan/PVOH composite films with different BTCA contents are shown in Figure 2. The peak value of the composite film at 3278 cm^−1^ was attributed to the stretching vibration of O–H in xylan and PVOH. Compared with the theoretical absorption peak (3600 cm^−1^) [39] of O–H, the frequency of stretching vibration of O–H was lower, indicating hydrogen bond interaction between xylan and PVOH. An obvious stretching vibration absorption peak of alkane C–H at 2906 cm^−1^ and a bending vibration absorption peak of C–O bond at 1035 cm^−1^ were clearly observed. The absorption peak of BTCA at 1689 cm^−1^ [40] was attributed to the stretching vibration of C=O in the carboxyl group, while no absorption peak was observed near 1689 cm^−1^ in the pure xylan/PVOH composite film. For xylan/PVOH composite film with BTCA addition, a new absorption peak at 1715 cm^−1^ was observed, which corresponds to the stretching vibration of C=O in the ester bond [41]. With increasing BTCA addition, the stretching vibration absorption peak of C=O in the ester bond became more obvious. The results show that the carboxyl groups in BTCA were esterified and crosslinked with the hydroxyl groups in xylan/PVOH matrix.

### 3.2. Surface Morphology Analysis

**Figure 3:**
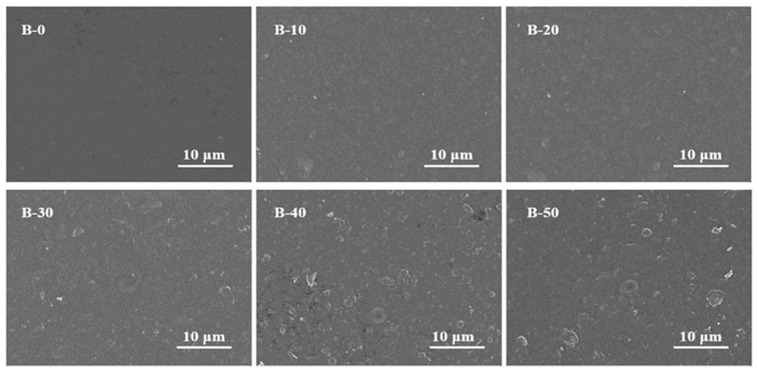
SEM images of BTCA-crosslinked xylan/PVOH composite films.

The surface morphology of the BTCA-crosslinked xylan/PVOH composite film was observed by SEM, and the results are shown in Figure 3.

Pure xylan/PVOH composite film has a smooth surface, although a few pores on the surface of the film can be observed, indicating the good compatibility of xylan and PVOH. After adding BTCA, the surfaces of the B-10 and B-20 composite films were more compact than the surface of the B-0 film, without defects such as pores and cracks. This might be due to the esterification of BTCA with xylan and PVOH, which helped xylan and PVOH to form a stable crosslinking structure [42]. With increasing BTCA loading, the surface roughness increased. At BTCA loading of greater than 30%, a granular substance appeared on the surface of the composite film, and the excessive addition of BTCA led to the increase in the surface roughness of the film. The four carboxyl groups of BTCA reacted selectively with hydroxyl group of hemicellulose and PVOH. Xylan and PVOH were not completely crosslinked, resulting in easy sliding between xylan and PVOH molecular chains, which increased the surface roughness and even led to micropore formation.

### 3.3. XRD Analysis

**Figure 4:**
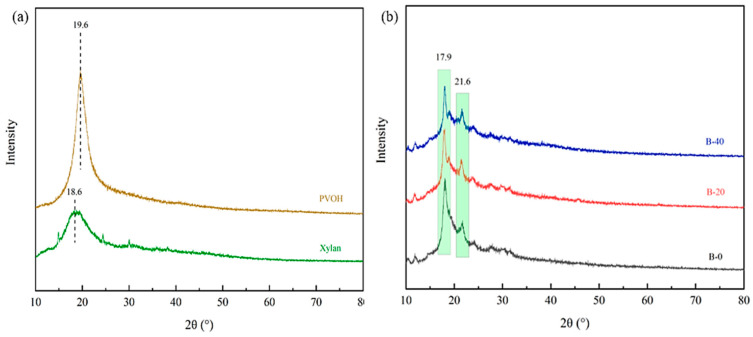
XRD patterns of PVOH and xylan (**a**) and BTCA-crosslinked xylan/PVOH composite films (**b**).

The X-ray diffraction pattern of the BTCA-crosslinked xylan/PVOH composite film is shown in Figure 4. The characteristic diffraction peak of xylan is located at 2*θ* = 18.6°, and the characteristic diffraction peak of PVOH is located at 2*θ* = 19.6°. The characteristic diffraction peak of xylan in the xylan/PVOH composite film shifted from 2*θ* = 18.6° to 2*θ* = 21.6°, and the characteristic diffraction peak of PVOH shifted from 2*θ* = 19.6° to 2*θ* = 17.9°. This may be due to the change in crystal structure of the two polymers through hydrogen bonding during the formation of the hemicellulose composite film [43]. The wide diffraction peaks of the B-0 film at 2*θ* = 17.9° and 21.6° indicated that xylan/PVOH matrix was semi-crystalline. After the addition of BTCA, no new crystallization peaks appeared in the xylan/PVOH composite films, but the diffraction peaks of the B-20 and B-40 films decreased at 2*θ* = 17.9° and 21.6° compared with the B-0 film. PVOH and xylan are semi-crystalline due to the hydroxyl group on the side chain [44]. When BTCA in the B-20 and B-40 composite films were esterified with the hydroxyl group of the film matrix [45], a crosslinked structure was formed inside the film and the number of hydroxyl groups in the xylan and PVOH was reduced, thus reducing the crystallinity and increasing the amorphous region of the film.

### 3.4. Surface Wettability

In order to evaluate the hydrophobicity of the chemically modified xylan/PVOH film, the surface contact angle of the BTCA-crosslinked xylan/PVOH composite film was measured using a contact angle measuring instrument. Five different positions on each sample were tested, and the average value was used as the static contact angle of the sample. The surface hydrophobicity of packaging materials is usually studied via the measurement of the water contact angle, and a film surface with a contact angle greater than 90° can be regarded as hydrophobic surface [46].

**Figure 5:**
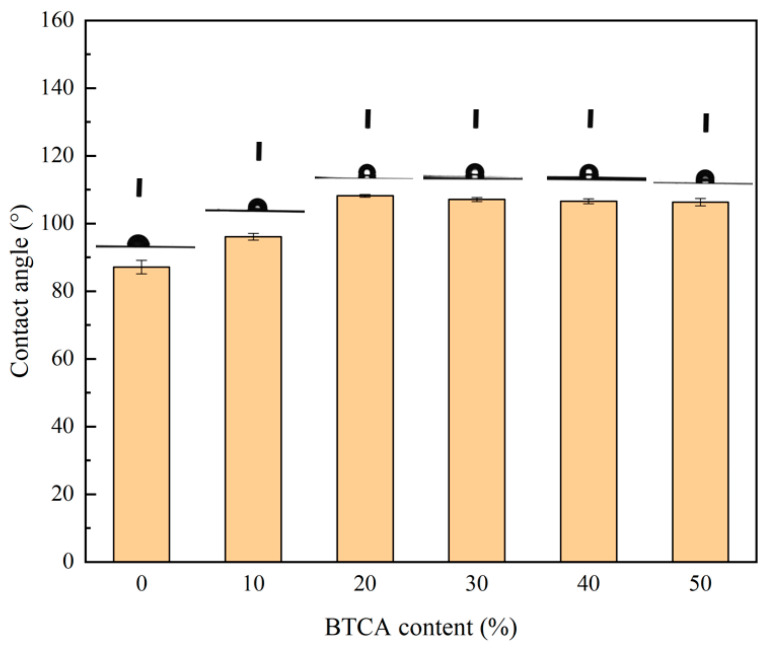
Contact angle results of BTCA-crosslinked xylan/PVOH composite films.

As shown in Figure 5, the contact angles of B-0, B-10, B-20, B-30, B-40 and B-50 were 87.1°, 96.1°, 108.2°, 107.1°, 106.6° and 106.3°, respectively. With the increase in BTCA content, the static contact angle of the composite film increased, and the BTCA-crosslinked xylan/PVOH composite film was considered hydrophobic. With increasing BTCA loading from 0 to 20%, and the surface contact angle of the composite film increased from 87.1° to 108.2°, an increase of 24%. This was mainly due to the esterification of the carboxyl group in BTCA with the hydroxyl group in the xylan/PVOH matrix, which reduced the effective number of hydrophilic hydroxyl groups in the composite film matrix and increased the exposure of hydrophobic groups on the film surface, thus improving the hydrophobicity of the hemicellulose-based composite film [28]. When the BTCA content was further increased, the static contact angle of the composite film began to decrease. Compared with the B-20 composite film, the contact angle of the B-50 film decreased from 108.2° to 106.3°, which was due to the high content in BTCA of many unreacted hydrophilic carboxyl groups, which were exposed on the film surface, meaning that the improvement effect of BTCA on the hydrophobic properties of xylan/PVOH composite films gradually weakened [47].

### 3.5. Mechanical Properties

**Figure 6:**
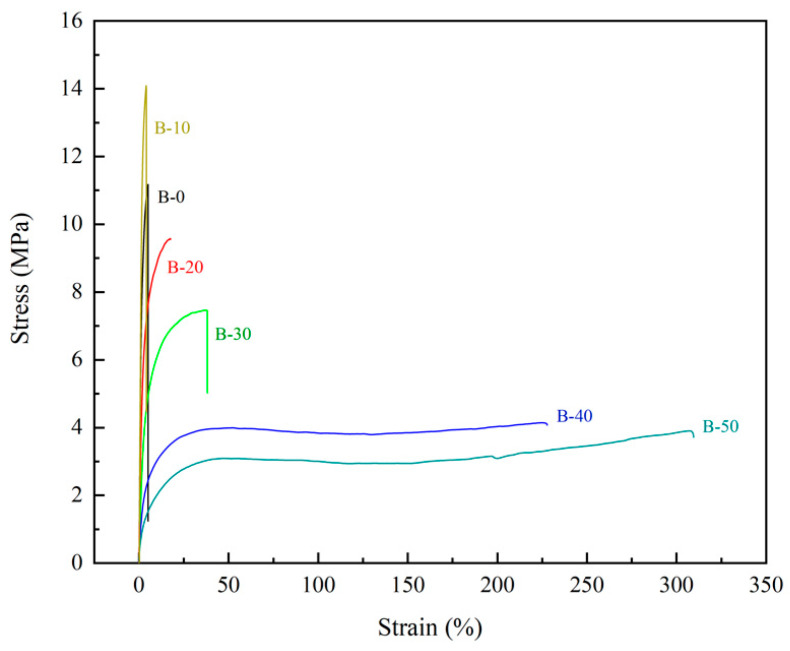
Typical tensile–strain curves of BTCA-crosslinked xylan/PVOH composite films.

The test results of mechanical properties of the BTCA-crosslinked xylan/PVOH composite films are shown in Table 2 and Figure 6. It is seen that with increasing BTCA loading, the tensile strength of the composite film increased at first and then decreased, while the elongation at break slightly decreased at 10% BTCA loading, but drastically increased at higher BTCA loadings. More specifically, it can be seen from Table 2 that the tensile strength of the B-10 film increased from 11.19 MPa (for blank sample) to 13.99 MPa at 10% BTCA loading. The stiffness of the film was enhanced, and the maximum Young’s modulus reached 1014 MPa. This was due to the formation of a dense network structure, resulting in enhanced intermolecular force and increased tensile strength of the composite film [48]. At higher BTCA loadings, the tensile strength and Young’s modulus of the composite film began to decrease and the elongation at break increased significantly. At BTCA loadings of higher than 20%, the elongation at break of the composite film increased significantly. The elongation at break of the B-50 film reached 314.28%, while the tensile strength decreased significantly. This was due to the selective reaction between the four carboxyl groups of excess BTCA and the hydroxyl group of the composite film matrix, resulting in an increase in the flexibility of the molecular chain and a decrease in the crosslinking degree of the system. In addition, the small residual molecules of BTCA also played a plasticizing role [39,49], which increased the interstitial volume of the material and promoted the fluidity of xylan and PVOH molecular chains, resulting in better flexibility and lower tensile strength of the composite film.

### 3.6. Oxygen Barrier Properties

In the food packaging industry, the oxygen permeability of packaging materials is a key factor to determine the shelf life of food. Low oxygen permeability is required of packaging material.

The oxygen permeability of xylan/PVOH composite films with different BTCA contents at 23 °C and under 50% relative humidity, together with that of some typical packaging materials, is shown in Table 3. It can be seen from the table that, compared with the unmodified xylan/PVOH composite film, all hemicellulose-based composite films modified with BTCA have lower oxygen permeability. The composite film with 20% BTCA loading has the lowest oxygen permeability of 0.43 (cm^3^·µm)/(m^2^·d·kPa), 79.6% lower than that of the unmodified xylan/PVOH film. This value is also lower than those of citric acid crosslinked hemicellulose film (5.4 cm^3^·µm·m^−2^·d^−1^·kPa^−1^) [47], sodium trimetaphosphate crosslinked hemicellulose film (3.98 cm^3^·µm·m^−2^·d^−1^·kPa^−1^) [28] and ethylene-vinyl alcohol copolymer (EVOH, 2.88 cm^3^·µm·m^−2^·d^−1^·kPa^−1^) [50], indicating that BTCA-crosslinking is effective in barrier property enhancement. The reduced oxygen permeability of the composite film was mainly due to the esterification and crosslinking reaction between BTCA and xylan/PVOH matrix. With the increase in crosslinking degree, the distance between xylan and PVOH molecular chains became smaller. The decreased chain mobility made the composite film more compact and enhanced its ability to hinder oxygen diffusion. At the same time, the free volume inside the film became smaller, and it was more difficult for oxygen to enter the composite film matrix, thus reducing the oxygen permeability [51]. When the content of BTCA was further increased from 20%, the oxygen permeability of the composite film began to increase. This change may be due to the incomplete crosslinking reaction between the excess BTCA and the hydroxyl groups in the composite film matrix, which had a plasticizing effect [47], resulting in an increase in the gap between the molecular chains of the film matrix and some micropores on the film surface. Therefore, oxygen permeated more easily, demonstrating the increased oxygen permeability of the composite film.

### 3.7. Thermal Stability

**Figure 7:**
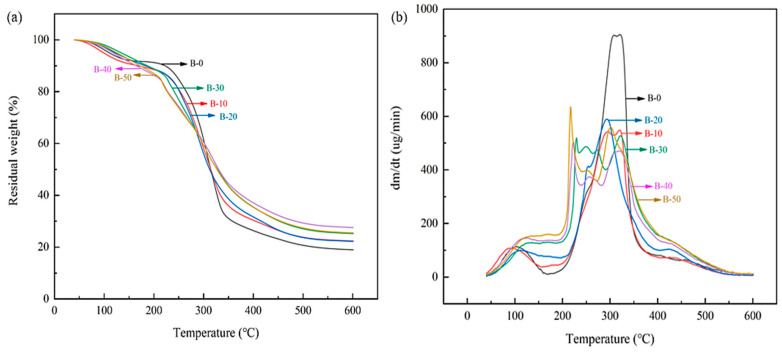
Thermogravimetric (TGA) curves (**a**) and derivative thermogravimetry (DTG) curves (**b**) of BTCA-crosslinked xylan/PVOH composite films.

The thermal stability of the BTCA-crosslinked xylan/PVOH composite film in the range of 40 °C to 600 °C was studied by thermogravimetric analyzer (TGA). As shown in Figure 7, the film has three main stages of weight loss. The weight loss in the first stage (60–210 °C) is mainly caused by water vapor evaporation (both internal water and that on adsorbed on film surface). In the second stage (210–340 °C), the weight loss is the largest, which is mainly due to the degradation of the side chains of beech xylan and PVOH and the degradation of small-molecule BTCA. Weight loss in the third stage (340–520 °C) is due to the carbonization of the polymer. The C–C main chains of xylan and PVOH are broken in this temperature range.

When BTCA was added to the xylan/PVOH mixed matrix, the thermal decomposition rate of the BTCA-crosslinked xylan/PVOH composite film decreased in the second stage, indicating that the thermal stability of the composite film was improved. The decrease in the thermal decomposition rate in the second stage can be explained by the esterification and crosslinking reaction of BTCA with xylan and PVOH. The crosslinking network structure required more energy to break the molecular chain [54]. At BTCA loading of over 30%, another peak at around 220 °C appeared in the thermal decomposition curve, which is mainly related to the decomposition of excess BTCA and the side chain breakage of xylan and PVOH.

## 4. Conclusions

In summary, the preparation of a hemicellulose-based composite film by crosslinking xylan and PVOH with the esterifying agent BTCA is a low-cost, simple and green method. In the presence of phosphorus-free catalyst sodium propionate, BTCA reacted with xylan and PVOH to form a close crosslinking structure, which improved the hydrophobicity and barrier performance of the composite film. When the content of BTCA was 10%, the formation of crosslinking structure increased the tensile strength of the composite film from 11.19 MPa to 13.99 MPa. With the increase in BTCA content from 20% to 50%, BTCA mainly played a plasticizing role in the composite film, significantly improving the flexibility of the composite film. At 20% BTCA content, the maximum contact angle of 108.2° and the minimum oxygen permeability of 0.43 (cm^3^·µm)/(m^2^·d·kPa) were obtained, and the oxygen barrier performance of the composite film was comparable to that of EVOH, a material with good barrier properties. The thus prepared xylan/PVOH composite film with good hydrophobicity and oxygen barrier properties shows promise as an alternative or substitute for traditional petrochemical-based food packaging materials.

## Figures and Tables

**Table 1:** Nomenclature and composition of films.

Sample	BTCA Mass Fraction (%)	Mass of Xylan (g)	Mass of PVOH (g)	Mass of Sorbitol (g)
B-0	0	1.8	0.6	0.6
B-10	10	1.8	0.6	0.6
B-20	20	1.8	0.6	0.6
B-30	30	1.8	0.6	0.6
B-40	40	1.8	0.6	0.6
B-50	50	1.8	0.6	0.6

**Table 2:** Tensile test results of BTCA-crosslinked xylan/PVOH composite films.

Sample	Thickness (µm)	Tensile Strength (MPa)	Elongation at Break (%)	Young’s Modulus (MPa)
B-0	126 ± 5	11.19 ± 0.85	5.24 ± 0.99	823 ± 69
B-10	129 ± 3	13.99 ± 0.53	4.86 ± 1.16	1014 ± 112
B-20	133 ± 6	10.02 ± 0.79	17.62 ± 1.73	551 ± 19
B-30	137 ± 5	7.78 ± 0.72	37.1 ± 1.59	189 ± 23
B-40	144 ± 7	4.23 ± 0.36	227.28 ± 6.73	109 ± 18
B-50	145 ± 6	3.89 ± 0.41	314.28 ± 4.12	96 ± 6

**Table 3:** Comparison of oxygen permeability of the packaging films in the literature and this work.

Sample	Oxygen Permeability [(cm^3^·µm)/(m^2^·d·kPa)]	Reference Citation
B-0	2.11 ^1^	-
B-10	0.57 ^1^	-
B-20	0.43 ^1^	-
B-30	0.78 ^1^	-
B-40	1.52 ^1^	-
B-50	1.98 ^1^	-
TiO_2_-KH550 nanoparticle-reinforced PVA/xylan composite films	4.01	[12]
Citric acid crosslinked hemicellulose films	5.4	[47]
Sodium trimetaphosphate crosslinked hemicellulose films	3.98	[28]
Cellulose nanofibril (CNF) films	72	[52]
Low-density polyethylene (LDPE)	772	[53]
High-density polyethylene (HDPE)	237	[53]
Ethylene vinyl acetate (EVA)	1431	[53]

^1^ Test conditions: 23 °C, 50% RH.

## Data Availability

Not applicable.
